# Meat quality characteristics of pork bellies in relation to fat level

**DOI:** 10.5713/ab.20.0612

**Published:** 2021-01-01

**Authors:** Van-Ba Hoa, Kuk-Hwan Seol, Hyun-Woo Seo, Pil-Nam Seong, Sun-Moon Kang, Yun-Seok Kim, Sung-Sil Moon, Jin-Hyoung Kim, Soo-Hyun Cho

**Affiliations:** 1 National Institute of Animal Science, Rural Development Administration, Wanju 55365, Korea; 2 Sunjin Meat Research Center, Ansung 17532, Korea

**Keywords:** Eating Quality, Fat Level, Flavor, Pork Belly, Technological Quality

## Abstract

**Objective:**

Pork belly is considered as the most commercially important and preferable primal cut by consumers worldwide. Thus, this study was conducted to determine the effects of fat levels on the meat quality characteristics of pork bellies.

**Methods:**

Seventy-eight growing-finishing pigs collected from different commercial pig farms were slaughtered and used in the present study. After slaughter 24 h, bellies were fabricated according to the Korean Pork Cutting Specification, and immediately sampled for analysis of their fat content. Based on the fat levels, the bellies were segregated into three different groups: low fat (LF, fat ≤20%, n = 15), medium fat (MF, fat 21% to 30%, n = 30), and high fat (HF, fat ≥31%, n = 33). The bellies were then analyzed for meat quality traits, fatty acids, flavor compounds and eating quality properties.

**Results:**

The HF group had lower moisture and cooking loss levels compared to the other groups (p<0.05). The LF group presented higher proportions of polyunsaturated fatty acids compared to the other groups (p<0.05). The LF group showed higher amounts of the Maillard reaction-derived flavor compounds (e.g., 2,5-dimethyl pyrazine, 2-ethyl-3,5-dimethyl, and 4-methylthiazole) associated with meaty and roasty flavors whereas, the HF group showed higher amounts of oleic acid-derived compounds (e.g., nonanal and octanal) associated with the fatty and oily flavors. Interestingly, significantly higher scores for all the eating quality attributes (flavor, juiciness, tenderness, and overall acceptance) were found in the HF group compared to those in the LF or MF group (p<0.05).

**Conclusion:**

The high-fat bellies (fat ≥31%) had a better technological quality and eating quality compared to the low-fat bellies (fat ≤20%). Thus, increasing the fat content may improve the technological quality and eating quality traits of pork bellies, however, this increase may also result in more trimmed loss due to excessively deposited body fat.

## INTRODUCTION

Among the main types of red meat, pork is the most consumed in the world, and the consumption of this meat type has incessantly increased over the years [1]. However, the demand for pork primal cuts distinctively differ depending on markets and cuisine cultures among countries [2,3]. Out of primal cuts, belly is the biggest cut accounting approximately 16% to 18% by weight in each pork carcass [4,5] and is considered as the most valuable cut which contributes a significant ratio to total pork carcass value [6]. The pork bellies generally have a high fat (HF) content (20% to 60%), low lean content (22% to 55%) and a quite complicate structure with multiple muscles and intermuscular fat layers [7]. Due to these typical features, for many years the bellies have not commonly been used in studies compared to other cuts like loin.

In general, pork bellies are highly preferable by consumers worldwide, and their market price therefore is several times higher compared to the all other remaining primal cuts in many markers [4]. A survey on the Asian’s pork consuming trend made by Choe et al [3] showed that most Chinese and Korean consumers show strongest preference for the belly followed by shoulder butt cut and loin cuts. The pork bellies are used as the main material for making popular grilled dishes [3] as well as for processing the other commercial meat products (e.g., bacon) that are the commercially important value-added meat product in the world [8]. Recently, due to the effects of breeding and feeding programs aimed at increasing lean content in pork carcasses, pork bellies have been become thinner, which causes an undesirable structure, high softness and lower yield of its processed products [9,10]. This implicates that the chemical composition (fat content and moisture etc.) may play an important role in determining the technological quality and eating quality of bellies and their processed products.

Till now, studies have found that fat content is one of the most important components affecting quality and sensory properties of *longissimus dorsi* muscles pork [11–13]. However, it still remains unknown how and whether the fat content affects the technological quality traits and sensory properties of pork belly. Because, all these studies have only used the loin muscles as the representative samples when studying the effects of pre-and post-harvest factors on pork quality [14–16]. It should be noted that a pork carcass with good quality loin doesn’t necessitate a high quality belly or other cuts, and thus drawing a conclusion on bellies quality based on the loin quality is inappropriate and misleading [16,17]. Since the final eating quality of pork bellies as well as their technological quality characteristics can be directly affected by the chemical composition such as fat content as mentioned above. There is a need to conduct studies aim at finding the factors that affect the quality of valuable cuts like belly. Therefore, the objective of this study was to investigate the effects of different fat levels on the technological quality traits, fatty acid profiles, volatile flavor compounds and sensorial properties of pork bellies.

## MATERIALS AND METHODS

### Animal care

The animal protocols used in the present study were reviewed and approved by the Institutional Animal Care and Use Committee (IACUC) at National Institute of Animal Science (approval number NIAS 20001992).

### Samples preparation

**Figure 1:**
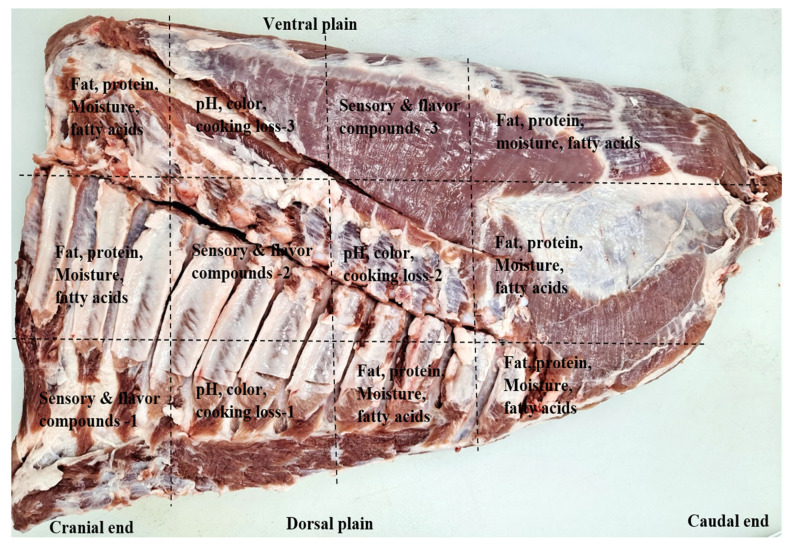
The representative diagram showing the sampling locations for pork bellies for the analyses.

A total of 78 crossbred ([Landrace×Yorkshire]♀×Duroc ♂) pigs at ages of about 175 to 190 days collected from different commercial pig farms were used in this study. Prior to slaughter, the pigs were fasted for 8 h but fully accessed to water. During the investigating period, numerous slaughter batches were carried out at one week intervals. The slaughter was carried out at an abattoir (Jeonju, Korea) following the industry-accepted procedures of Korea Institute of Animal Products Quality Evaluation (KAPE) [18]. All carcasses were then split, hanged and cooled for 24 h before fabrication. The carcasses were fabricated into 7 primal cuts such as; loin, belly, hind and fore legs, shoulder butt, tenderloin and shoulder rib according to the guidelines of Korean Pork Cutting Specification [19]. All bellies were collected from the left side of the carcasses (between 4th and 5th thoracic vertebrae with a straight cut perpendicularly to the axis of the carcasses), and their weights were recorded after removing skin, individual ribs and rest of ventral part (e.g., milk glands). The information (e.g., live weight, carcass weight, total trimmed fat, skin and bone, and belly weights) regarding the used animals is shown in Table 1. To minimize the quality variations caused by different anatomical locations, each belly was cut into three sections (dorsal, central, and ventral). Each section was then cut into sub-samples depending on each analysis as shown in Figure 1. On each the section of each belly, all measurements of meat quality (fat content, pH color, cooking loss, flavor compounds, fatty acid composition and sensory properties) were carried out, and the mean value for each analytical parameter was computed by averaging the values obtained from the three sections. Immediately after the samples preparation, the sub-samples in three sections of each belly were combined together, ground, well-mixed and used for crude fat content determination. The fat content was determined following the AOAC Official Methods 2007.04 by using a Food Scan Lab 78810 (Foss Tecator Co., Ltd., Hilleroed, Denmark) as described by Anderson [20]. Considering the range of fat content, the bellies were segregated into three different groups: low fat (LF; fat ≤20%, n = 15), medium fat (MF; fat 21% to 30%, n = 30), and HF (fat ≥31%, n = 33). The sub-samples of the bellies were then assigned into their corresponding fat level groups and used for meat qualities analysis. Analysis of proximate composition, color and pH, were performed on fresh samples (the sampling day), while vacuum packed in oxygen impermeable polyethylene bags and storage frozen (−20°C) samples were used for analysis of fatty acid composition, flavor compounds and sensory evaluation.

### Proximate composition

The contents of moisture, protein and collagen were measured following the AOAC Official Methods 2007.04 by using a Food Scan Lab 78810 (Foss Tecator Co., Ltd., Denmark) as described by Anderson [20]. Each sample was determined in triplicates.

### pH measurement

The pH of the belly samples was measured using a pH*K 21 (NWK-Technology GmbH, Kaufering, Germany) equipped with a stainless steel and solid-state probe after calibrating with pH 4.0 and 7.0 standards (NWK Technology., Germany). The pH values were obtained by inserting the solid-state probe of device deeply into the muscle tissues on three different lean layers. Care was taken to avoid measuring on the fat layers.

### Instrumental color and cooking loss measurement

The measurements of the surface color and cooking loss were carried out on a same 2.5 cm-thick steak (3 steaks from 3 different anatomical sections per belly). Following a 30 min blooming period at 4°C, the color was measured on the three freshly-cut surface locations of the steaks using a Minolta Chroma Meter CR-400 with a D65 illuminant*1 and 2° observer (Minolta Camera Co, Osaka, Japan). Before using, the Chromameter was standardized against a white tile (Y = 86.32, X = 0.3165, and y = 0.3242). The measurement was carried out under white lighting and the results were reported as CIE L* (lightness), CIE a* (redness), CIE b* (yellowness), chroma and hue angle (h°).

After the color measurement was completed, the belly steaks (approximately 150 g each) were placed into individually pre-labeled plastic bags and cooked in a pre-heated 72°C water bath until their core temperature reached 70°C. A copper-constantan thermocouple attached to a Thermo recorder (Model TR-71U; T & D Corp., Tokyo, Japan) was used to monitor the core temperature of the samples. When reaching the targeted temperature, the cooked samples were immediately removed from the water bath and then cooled down for 30 min under running water. The weights of the cooked samples were recorded after removing the plastic bags and surface water absorbed with wiping papers. The cooking loss was calculated as the weight loss percentage during cooking.

### Fatty acid profiles analysis

The sub-samples from sections in each the belly were combined, comminuted, well-mixed and used for fatty acids analysis. The lipids in the samples were extracted using a solvent mixture of chloroform: methanol (2:1, v/v) and then were converted to fatty acid methyl esters (FAME) as described in our previous study [14]. Approximately 1.0 mL of FAMEs were transferred to auto-sampler vials and was sealed. The separation of FAMEs was achieved using a gas chromatography/flame ionization detector (GC-FID, Varian Technologies, Palo Alto, CA, USA) equipped with an Omegawax capillary column (30 m×0.25 mm×0.25 μm film thickness; Supelco, Bellefonte, PA, USA). The GC oven temperature was maintained at 50°C for 1 min, and ramped at a rate of 25°C/min to 200°C, and further raised at a rate of 5°C per min to 230°C.

The injection port and detector temperatures were set at 250°C and 260°C respectively. Identification of fatty acids in samples was carried out by comparing their retention times with those obtained from standard fatty acids. Individual fatty acids were expressed as relative percent (%) of total fatty acids FAMEs.

### Sensory evaluation

The sensory evaluation of the belly samples was performed using trained panels. The panels comprised 8 trained members who were institutional staff. Given the experimental design for sensory evaluation, 3 sub-samples from the sections (dorsal, central, and ventral planes) for each the belly were assessed. Each of them was separately evaluated by six panelists. After defrosting for 2 h in a cooling room (4°C), 7 slices (50 mm×50 mm×4 mm) were manually prepared from each the sub-sample. Out of them, one was used for the overall color evaluation after 30 min blooming. The rest of 6 strips were cooked at around 180°C on an open tin-coated grill for about 2 min and turned every 30 s. The cooking temperature was monitored using an infrared thermometer (model: DT8380, Shenzhen, China). Immediately after cooking, the samples were placed on individual dishes and served to the panelists who then tasted for flavor, juiciness, tenderness and overall acceptability using a 7-point hedonic scale (7 = extremely like; 6 = like very much; 5 = like moderately; 4 = neither like nor dislike; 3 = dislike moderately; 2 = dislike very much; and 1 = dislike extremely) as described by Meilgaard et al [21]. After evaluating each sample, the panelists were asked to refresh their palate with drinking water and unsalted crackers. All sensory assessments were conducted in the sensory panel booth room equipped with white lighting.

### Volatile flavor compounds analysis

Volatile flavor compounds in the cooked bellies were extracted and then analyzed using the protocols developed by Ba et al [22]. Briefly, following cooking (the cooking conditions were same as those used in the sensory evaluation), each cooked sample (2.0 g) was immediately taken and placed into a 20-mL headspace vial and sealed with PTFE-faced silicone septum. The vial containing samples were then extracted for volatiles using a solid phase micro-extraction (SPME) device with a 75 μm carboxen/polydimethylsiloxane (CAR/PDMS) fibre (Supelco, USA). The extracting process was carried out at 65°C for 60 min using a fully automated SPME sample preparation instrument (Model: AOC-5000 Plus, Agilent Technologies, Santa Clara, CA, USA). The volatiles were analyzed using a GC (model: 7890B) with mass spectrophotometry (MS, model: 5977B MSD, Agilent Technologies, USA) equipped with a capillary column (30 m×0.25 mm× 0.25 μm film thickness) (Agilent J & W Scientific, Folcom, CA, USA). The separation of the volatiles was performed under the conditions that were same as those described in the above cited literature [22]. The volatiles were identified by comparing their mass spectra with those in the Wiley library (Agilent Technologies, USA) and/or by matching their retention times with those of external standards. The identified compounds were quantified by comparison its corresponding peak area with that of an internal standard (1.0 μL of 2-methyl-3-heptanone, 816 mg/mL in methanol). The results were expressed as μg/g cooked meat.

### Statistical analysis

The obtained data was subjected to statistical analysis using a Statistic Analysis System (SAS) package (SAS Institute, Cary, NC, USA, 2007). The data were analyzed by using the analysis of variance procedure of the SAS. In the statistical model, the fat level group was considered as the fixed effect while, the meat quality traits, fatty acids, flavor compounds and sensory attributes were considered as the dependent variables. Means were compared using Duncan’s multiple range test. Significance was defined at p<0.05. Pearson’s correlation coefficients were calculated between the above mentioned traits with fat level groups.

## RESULTS AND DISCUSSION

In many countries, pigs are usually finished at around 170 to 200 days old and body weights of 110 to 140 kg for commercial meat production [11,16,23]. In the present study, the bellies were collected from the pigs with finishing ages of 175 to 190 days which resulted in significant (p<0.05) differences in live weight, dressing percentage, and yields of carcass compositions (Table 1). The live weight and total fat yield (fats trimmed from internal organs and primal and sub-primal cuts) were significantly higher in the HF group followed by the MF and LF groups (p<0.05). The yield of bellies was also higher in the HF group compared to that of the LF group (p<0.05). Especially, no differences occurred in the dressing percent among the three groups studied (p>0.05). These results imply that increasing the finishing weight led to an increased belly yield but did not affect the dressing percentage. Aligning with the present results, Correa et al [24] reported that yield of commercial pork belly significantly increased with increased slaughter weight.

### Proximate composition and technological quality traits of bellies as affected by fat level

The results regarding the proximate composition and technological quality traits are summarized in Table 2. All the chemical compositions including fat (intermuscular, intramuscular and subcutaneous fat), moisture, protein and collagen under study were significantly affected by the fat level (p<0.05). The average fat content was 19.37%, 26.29%, and 32.87% for the LF, MF, and HF groups, respectively. These fat levels generally were lower than the level (46%) reported by Soladoye et al [23] for bellies sampled within 15 cm of cranial end, and also were lower than levels (31.5%, 46.6%, and 64.4% for ventral, central and dorsal sections, respectively) reported by Trusell et al [9] for pork bellies. These contrasting results may be linked to the slaughter weights difference; in their studies the bellies were collected from heavier pigs (130 to 140 kg) which may be associated with increased fat deposition in carcasses [25]. Unlike the fat content, the moisture content was the lowest in the HF group, followed by those in the LF and MF groups (p<0.05). This moisture content generally was lower than the value (41.3%) reported by Soladoye et al [23] for higher-fat bellies. Thus, we observed that the bellies of the HF group had a lower moisture content and vice versa, which agrees with the general rule that fat and moisture contents in meat are inversely related with each other [26].

Regarding the meat color, it was observed that no differences occurred in the L* (lightness), a* (redness) and chroma mean values among the three groups (p>0.05). Only two traits that differed across the groups were b* (yellowness) and hue angle in which the LF group showed a lower b* value and the MF group had a higher hue angle value compared to the other groups (p<0.05). Compared with our data, Knecht et al [16] reported lower L* values (49.54 to 50.96) but higher a* values (16.83 to 18.96) for the same anatomical locations of pork bellies. This could be related to the genetics and slaughter weight differences between the studies. In term of technological quality traits, the fat level group did not affect the meat pH but did significantly affect the cooking loss; the cooking loss significantly decreased with increased fat level (p<0.05). The level of cooking loss ranging from 16.16% to 20.08% among the fat level groups in this study were generally lower than values (25% to 40%) reported by Knecht et al [16] for pork bellies. This may be related to the lower pH values (5.57 to 5.66) of the belly samples in their study, since the water holding capacity determined by cooking loss or drip loss is inversely correlated to meat pH [27].

### Fatty acid profiles of bellies as affected by fat level

The fatty acid compositions in the bellies from the three fat level groups are presented in Table 3. For the saturated fatty acids (SFA), we observed that the proportions of myristic acid (C14:0), palmitic acid (C16:0) and stearic acid (C18:0) as well as total SFAs content were similar in all the groups (p>0.05). This signifies a similar saturation degree of the bellies or a similar *de novo* SFAs synthesis and the exogenous SFAs uptake from diet of pigs in all the groups studied. In contrast to the SFAs, the fat level showed its particular effects on the unsaturated fatty acids (UFAs) of the bellies. The most predominant monounsaturated fatty acid (MUFA) found was C18:1n9 (oleic acid) whose proportion was the highest in the HF group compared to that in the LF group (p<0.05). Inversely, the proportions of almost all polyunsaturated fatty acids (PUFA) such as linoleic acid (C18:2n6) and linolenic acid (C18:3n3), arachidonic acid (C20:4n6) and docosatetraenoic acid (C22:4n6) as well as total PUFAs content were significantly higher in the LF group compared to the other groups (p<0.05). These observations could be attributed to the higher intensity of *de novo* PUFAs synthesis and/or the exogenous uptake of these PUFAs from diet by the pigs from this group. Regarding this, Correa et al [24] noted that leaner pork bellies are associated with higher proportions of UFAs when compared to fatter bellies from fast growing pigs. Compared with our data, those of Trusell et al [9] reported higher proportions of C18:2n6 (15% to 18%) and total PUFAs (17% to 20%) in pork bellies finished at heavier weight. However, the proportion (38% to 39%) of C18:1n9 reported by these authors was almost similar to the level in the HF group but higher than the levels in the LF and MF groups. Otherwise, total PUFAs content reported by Correa et al [28] for pork bellies was similar to the level in the LF group but higher than the levels in the HF and MF groups.

Fatty acid profiles in meat not only reflect the nutritional value but also strongly determine the development of flavor characteristics of cooked meat [29]. A positive correlation existing between MUFAs, mainly oleic acid, with “fatty” flavor of cooked meat has been reported by Okumura et al [30]. Whereas, high proportions of PUFAs (e.g., C18:3n-3) in meat may cause off-flavors of cooked meat [29]. On the other hand, the LF group showed higher PUFA:SFA ratio compared to that of HF group (p<0.05). From a point of view of nutritional value, compared with the MF or HF groups the bellies of the LF group generally exhibited a healthier fatty acid profiles because it showed the PUFA:SFA and n6:n3 ratios that were closer to the recommended values for human diets [31]. Overall, it may be said that fat level partly affected the fatty acid compositions which may have certain effects on the flavor development during cooking as well as nutritional values of bellies.

### Volatile flavor compounds of cooked bellies as affected by fat level

A total of thirty compounds comprising 13 aldehydes, 6 alcohols, 5 hydrocarbons, and 4 nitrogen-and sulfur-containing compounds and 2 furans were identified from the three groups (Table 4). It was observed that the fat level showed a certain effect on the volatile flavor profiles, mainly on the lipid oxidation/degradation-derived compounds (e.g., aldehydes), followed by the products (e.g., sulfur-and nitrogen-containing compounds) produced from the Maillard reaction between amino acids with reducing sugars. All the three groups exhibited their volatile flavor profile characteristic of high-fat cut since almost all of the compounds were likely produced from the oxidation/degradation of UFAs [32]. Aldehydes were the most predominant class in all the groups. Out of them, six compounds including: 2-ethyl hexanal, 3-methy butanal, 2-methyl butanal, hexanal, octanal and nonanal showed significant differences among the groups (p<0.05). 3-Methy butanal, 2-methyl butanal have been found to be formed from the Strecker degradation of amino acids such as isoleucine and leucine, respectively [33]. Interestingly, the amounts of these two aldehydes were significantly higher in the LF groups compared to the MF or HF groups (p<0.05). This could be related to the higher protein content in the LF group (Table 2). Researchers have reported that the 3-methyl-butanal and 2-methyl-butanal confer the desirable flavors such as meaty fishy, nutty and onion notes in cooked meat [34–37]. Hexanal is known to be formed from the degradation/oxidation of linoleic acid [29,33], and was the most predominant compound in all the groups. However, a significantly (p<0.05) higher amount of this compound was found in the bellies of the LF group compared to the MF or HF group, and this may be related to the higher proportion of linoleic acid in the LF group (Table 3). Hexanal may produce undesirable flavors (e.g., grass and rancid odors) in cooked meat at higher concentrations [35]. In contrast, the amount of octanal was significantly higher in the HF group compared to the LF group (p<0.05). Also, the concentration of nonanal was higher in the HF group followed by the MF and LF groups (p<0.05). Octanal and nonanal are two the most important oleic acid-derived compounds [33] which confer the pleasant flavors such as fruity, fatty, sweet and oily odors in cooked meat [35–37].

The alcohols are important aroma compounds for development of cooked meat flavors [32]. It was observed that all the identified alcohols were not different among the fat level groups studied except the 1-heptanol whose amount was significantly (p<0.05) higher in the HF group compared to the LF or MF groups. 1-heptanol originates from oleic acid oxidation in meat during cooking [33], and has also been reported in pork *longissimus dorsi* muscles [11]. In cooked meats, hydrocarbons are formed from the lipid oxidation/or amino acids Strecker degradation, and they contribute little to the development of cooked meat flavors due to their high odor-detection threshold [32]. Our results depict that none of the identified hydrocarbons showed differences among the three groups (p>0.05).

It is well known that the products (e.g., sulfur- and nitrogen-containing compounds) formed through the Maillard reaction between amino acids and a reducing sugar, are important to the cooked meat flavors development [32]. Our results depict that all the Maillard reaction-derived products showed differences among the groups, except carbon disulfide. Particularly, the amounts of 2,5-dimethyl pyrazine and 2-ethyl-3,5-dimethyl were significantly higher in the LF groups compared to those in MF or HF group (p<0.05). These pyrazines have been reported to confer the desirable flavors such as; meaty, roasty and grilled odors of cooked meats [32, 37–39]. Similarly, the amount of another nitrogen-containing compound, 4-methylthiazole, was higher in the LF group compared to the HF group (p<0.05). This compound has been reported as a key odorant in cooked meat flavor (e.g., meaty and roasty odors) [32,39,40].

Overall, it appears that the bellies from the LF group exhibited higher amounts of the Strecker degradation-, Maillard reaction- and the PUFAs oxidation-derived flavor compounds which are associated with meaty and roasty flavors. Contrastingly, the bellies from the HF level group presented higher amounts of oleic acid-derived compounds which are associated with the fatty, sweet and oily flavors. The results indicating the differences in amounts of these flavor compounds are likely related to the variations in the content and nature of precursors (e.g., amino acids and fatty acids) present in bellies among the groups studied.

### Eating quality traits of cooked bellies as affected by fat level

Mean scores for the eating quality attributes of bellies in the three groups are presented in Table 5. On a 7-point hedonic scale, the bellies in all the groups were rated relatively high scores (above 5) for all the eating quality traits evaluated. Interestingly, it was observed that panelists gave significantly higher flavor, juiciness and tenderness scores for the bellies from the HF group than those from MF and LF groups (p< 0.05). A series of researches conducted to examine the effect of intramuscular fat (IMF) level on eating quality traits of pork (e.g., loin cut) has shown that increasing the IMF content resulted in increased flavor, juiciness and tenderness of meat [15,41]. This study for the first time, evaluated the eating quality attributes of bellies by the fat level, and the results indicating the considerably higher scores of eating quality traits for the HF group in comparison to the MF or LF groups is likely due to the positive effects of the fat content (e.g., IMF, intermuscular and subcutaneous layers). Regarding this, previous studies reported that fat content is the major source for generation of volatile flavor compounds in meat during cooking [29,32]. The significantly higher flavor score given for the HF group, therefore, is probably related to its higher amounts of MUFAs-derived flavor compounds (e.g., octanal and nonanal) associated with desirable odors (e.g., fatty and oily odors) (Table 4). On the other hand, a higher fat content may result in an increased amount of perceived moisture in muscle tissues, which improves the juiciness and tenderness of meat [42]. Aligning with the present results, Knecht et al [16] reported a similar trend; in a belly, sections from dorsal and central planes containing higher fat levels perceived by panelists to be juicier, more flavorful, palatable and acceptable than the leaner ventral sections. Furthermore, the bellies from the HF group also received the highest overall acceptance score (5.92) compared to the MF (5.55) and LF (5.42) group (p<0.05). The overall acceptability is the sum of all eating quality traits, therefore, the higher score in this group could be associated with the synergistic effects of the higher flavor, tenderness, and juiciness scores.

Furthermore, the relationship between the fat level groups with the selected meat quality traits, chemical composition and sensory attributes were also determined as shown in Table 6. There were significantly (p<0.05) positive correlations between the HF group with live weight (r = 0.830), dressing percentage (r = 0.843) belly yield (r = 0.835), C18:1n9 (r = 0.836, and all the eating quality traits; flavor (r = 0.953), juiciness (r = 0.933), tenderness (r = 0.956), and overall acceptance (r = 0.968). Whereas, the HF group was negatively correlated to moisture content (r = −0.930), protein (r = −0.879) and collagen (r = −0.911). In general, the results of Pearson’s correlation analysis were in line with those observed in the measurements of chemical composition and technological quality (Table 2), fatty acids (Table 3), and sensory quality (Table 5). Supporting the present finding, Wood et al [12], Ngapo et al [13], and Ba et al [11] also showed a positive correlation between IMF content with eating quality traits of pork *longissimus dorsi* muscle. Overall, pork bellies are considered as the most commercially important cut in a carcass, increasing the fat content (e.g., by prolonging fattening time or finishing at heavier weight), therefore, may improve their technological quality and eating quality attributes.

## CONCLUSION

The effects of fat levels on the quality traits of pork bellies were investigated in the present study. The higher the fat content the lower the moisture, collagen and cooking loss in the bellies and vice versa. The bellies with LF content (≤20%) exhibited a healthier fatty acid profiles indicating by higher proportions of PUFAs and PUFA:SFA ratio compared to the those containing higher fat content. The bellies with LF content had higher amounts of the Maillard reaction-derived flavor compounds associated with meaty and roasty flavors whereas, the bellies from the HF level group had higher amounts of MUFAs-derived compounds associated with the fatty and oily flavors. Noticeably, a better eating quality (e.g., flavor, juiciness and tenderness) was found in the HF group (≥31%) compared to that of the LF group (≤20%). Based on the results obtained in this study, it is concluded that high-fat bellies (≥31%) had a better technological quality as well as eating quality compared to the low-fat bellies (≤20%). Thus, it may be implied that increasing the fat content may improve the technological quality and eating quality traits of pork bellies, however, this increase may also result in more trimmed loss due to excessively deposited body fat.

## Figures and Tables

**Table 1:** The live weight and yields of carcass composition among the three fat level groups

Items	Fat level		

	Low fat	Medium fat	High fat
Live weight (kg)	109.35±7.19^c^	115.03±8.16^b^	118.76±8.72^a^
Dressing (%)	79.59±1.81^a^	80.01±1.26^a^	80.37±1.30^a^
Skin yield (%)	6.51±0.65^a^	6.56±0.87^a^	6.42±0.78^a^
Trimmed fat yield (%)	14.26±1.90^c^	16.98±3.47^b^	21.15±3.43^a^
Bone yield (%)	10.77±0.58^a^	10.40±0.88^ab^	9.96±0.74^b^
Belly yield (%)	13.50±0.62^b^	13.85±0.84^ab^	14.20±0.76^a^

a–c Means within a row in each cut with different letters are different at p<0.05.

**Table 2:** The proximate composition and technological quality traits of bellies as affected by fat level

Items	Fat level		

	Low fat	Medium fat	High fat
Proximate composition			
Fat (%)	19.37±1.49^c^	26.29±2.91^b^	32.87±5.37^a^
Moisture (%)	60.92±2.10^a^	56.43±2.72^b^	48.82±4.20^c^
Protein (%)	19.75±2.71^a^	17.31±1.64^b^	14.63±1.48^c^
Collagen (%)	3.46±2.39^a^	2.92±1.77^ab^	2.16±0.94^b^
Color traits			
CIE L* (lightness)	56.93±10.09	58.61±9.86	58.98±10.30
CIE a* (redness)	11.13±3.81	11.38±5.15	11.12±4.19
CIE b* (yellowness)	6.62±1.63^b^	7.01±3.42^ab^	7.48±1.78^a^
Chroma	13.08±3.70	13.70±4.93	13.28±4.16
Hue angel	32.35±9.23^b^	35.16±9.85^a^	34.19±9.76^ab^
pH	5.87±0.225	5.87±0.25	5.8±0.23
Cooking loss (%)	20.08±2.07^a^	17.288±3.23^b^	16.16±2.84^c^

a–c Means within a row in each cut with different letters are different at p<0.05.

**Table 3:** Relative proportion (%) of fatty acids in bellies as affected by fat level

Items	Fat level		

	Low fat	Medium fat	High fat
C14:0	2.17±0.08	1.63±1.15	1.73±0.62
C16:0	29.21±1.47	31.79±1.62	29.80±1.51
C16:1n7	2.34±0.68	2.33±0.33	2.34±0.23
C18:0	13.29±0.44	13.07±1.27	13.93±0.76
C18:1n9	36.79±1.66^b^	37.44±3.02^ab^	39.48±1.17^a^
C18:1n7	0.05±0.00	0.06±0.01	0.06±0.00
C18:2n6	14.36±0.92^a^	11.98±2.37^ab^	11.20±0.61^b^
C18:3n6	0.01±0.00	0.01±0.00	0.01±0.00
C18:3n3	0.64±0.06^a^	0.57±0.11^ab^	0.47±0.05^b^
C20:1n9	0.73±0.05	0.87±0.15	0.74±0.06
C20:4n6	0.27±0.02^a^	0.15±0.04^b^	0.16±0.03^b^
C22:4n6	0.13±0.02^a^	0.10±0.01^ab^	0.09±0.02^b^
SFA	44.66±1.63	46.49±1.48	45.45±1.67
UFA	55.34±1.63	53.51±1.48	54.55±1.67
Total MUFA	39.92±1.24	40.70±3.38	42.62±1.31
Total PUFA	15.42±0.98^a^	12.81±2.46^ab^	11.93±0.55^b^
Total n3 fatty acids	0.64±0.06	0.57±0.11	0.47±0.05
Total n6 fatty acids	14.78±0.92^a^	12.24±2.35^ab^	11.46±0.58^b^
n6/n3	22.98±0.72	21.45±0.40	24.74±3.68
MUFA/SFA	0.90±0.06	0.88±0.09	0.94±0.06
PUFA/SFA	0.35±0.03^a^	0.28±0.05^ab^	0.26±0.02^b^

SFA, saturated fatty acids; UFA, unsaturated fatty acids; MUFA, mono unsaturated fatty acids; PUFA, poly unsaturated fatty acids.

a,b Means within a same row with different superscripts are significantly different (p<0.05).

**Table 4:** Concentration (μg/g) of volatile aroma profiles in cooked bellies as affected fat level

Items	Retention time (min)	Fat level			Identify method^1)^	Odor descriptors from literatures	Source	References

		Low fat	Medium fat	High fat				
Aldehydes								
Propanal	1.723	0.04±0.01	0.04±0.01	0.06±0.01	MS+STD	Sweet, caramel and alcohol	Beef	[36]
2-ethylhexanal	2.167	0.03±0.01^a^	0.02±0.00^ab^	0.01±0.01^b^	MS+STD	NF		
3-methybutanal	2.72	0.05±0.01^a^	0.03±0.01^b^	0.02±0.01^b^	MS+STD	Meaty and fishy	Beef	[36,37]
2-methylbutanal	2.829	0.09±0.02^a^	0.05±0.01^b^	0.03±0.01^b^	MS+STD	Nutty and onion	Beef	[37]
Hexanal	6.121	3.12±0.15^a^	2.75±0.07^b^	2.56±0.05^b^	MS+STD	Green, fatty, fruity, aldehyde, and rancid	Beef, pork	[36,37]
Heptanal	9.261	0.17±0.03	0.13±0.03	0.16±0.01	MS+STD	Green, fatty, oily, oily, fatty, fruity, rancid and unpleasant	Beef, chicken	[37,38]
2-Heptenal	10.755	0.04±0.01	0.03±0.01	0.03±0.02	MS+STD	Soapy, fatty, almond, fishy and unpleasant	Beef	[35]
Benzaldehyde	10.873	0.04±0.01	0.05±0.00	0.06±0.01	MS+STD	Almond oil, bitter almond, burning aromatic taste	beef	[35]
Octanal	11.915	0.18±0.06^b^	0.20±0.03^ab^	0.29±0.05^a^	MS+STD	Sweet, fatty, fruity, lemon, green and oily	beef	[35,37]
Benzenacetaldehyde	12.874	0.01±0.00	0.01±0.00	0.02±0.01	MS+STD	NF		
E,2-octenal	13.19	0.02±0.01	0.02±0.01	0.02±0.00	MS+STD	Fatty, nutty and green	Beef	[35,36]
Nonanal	14.198	0.14±0.01^c^	0.20±0.00^b^	0.26±0.03^a^	MS+STD	Sweet, fat, fruity and citrus	Beef	[35,36]
E,2-nonenal	15.33	0.09±0.02	0.08±0.02	0.19±0.15	MS+STD	Fatty, aldehyde, nutty and cucumber	Beef	[35,36]
Alcohols								
1-penten-3-ol	3.067	0.01±0.00	0.01±0.00	0.01±0.00	MS+STD	NF		
4-amino-1-hexanol	3.302	0.19±0.02	0.24±0.02	0.23±0.04	MS	NF		
1-Pentanol	5.026	0.16±0.01	0.11±0.00	0.12±0.02	MS+STD	Roasted meat, mild odor, fruit	Beef	[35,38]
1-Heptanol	11.112	0.01±0.00^b^	0.01±0.00^b^	0.02±0.01^a^	MS+STD	Fragrant, woody, oily, green, fatty, winey, sap and herb	Beef	[35]
1-Octen-3-ol	11.356	0.09±0.02	0.05±0.02	0.09±0.05	MS+STD	Mushrooms	Beef	[35]
2-ethyl-1-hexanol	12.588	0.03±0.01	0.03±0.00	0.03±0.00	MS+STD	NF		
Hydrocarbons								
Toluene	4.929	0.01±0.00	0.01±0.00	0.01±0.00	MS+STD	NF		
1,3-dimethyl benzene	7.982	0.01±0.00	0.01.±.0.00	0.02±0.00	MS	NF		
Xylene	8.915	0.07±0.03	0.07±0.05	0.08±0.03	MS	NF		
2,4-dimethylhexane	13.029	0.03±0.01	0.03±0.00	0.03±0.00	MS	NF		
Benzoic acid	15.433	0.06±0.01	0.06±0.01	0.06±0.03	MS+STD	NF		
Sulfur- and nitrogen-containing compounds								
Carbon disulfide	1.862	0.01±0.00	0.01±0.00	0.01±0.00	MS	NF		
2,5-dimethyl pyrazine	9.558	0.03±0.00^a^	0.01±0.00^b^	0.02±0.00^b^	MS+STD	Roasty and toasty	Beef, pork	[32,39]
4-methylthiazole	11.475	0.23±0.01^a^	0.19±0.01^ab^	0.16±0.02^b^	MS+STD	Roasty and meaty	Beef, pork	[39,40]
2-ethyl-3,5-dimethylpyrazine	13.575	0.04±0.00^a^	0.02±0.00^b^	0.02±0.00^b^	MS	Roasty and toasty	Beef, pork	[32,37,39]
Furans								
2-pentylfuran	11.581	0.25±0.09	0.29±0.02	0.24±0.07	MS+STD	Green bean and butter	Beef	[35]
2-n-Octylfuran	17.889	0.04±0.02	0.03±0.00	0.02±0.01	MS+STD	NF		

NF, not found.

1) Identification method: the compounds were identified by mass spectra (MS) from library or external standard (STD).

a–c Means within a row with different letters are different at p<0.05.

**Table 5:** Mean scores (7-point scale) of sensory traits of bellies as affected by fat level

Group	Fat level		

	Low fat	Medium fat	High fat
Overall sensorial color	5.09±0.84	5.09±0.86	5.19±0.73
Flavor	5.36±0.75^b^	5.49±0.88^b^	5.78±0.81^a^
Juiciness	5.28±0.71^c^	5.47±0.79^b^	5.80±0.72^a^
Tenderness	5.09±0.78^b^	5.22±0.89^b^	5.52±0.82^a^
Overall acceptance	5.42±0.71^b^	5.55±0.82^b^	5.92±0.72^a^

The mean values were calculated using 7-point scale (7 = extremely like; 6 = like very much; 5 = like moderately; 4 = neither like nor dislike; 3 = dislike moderately; 2 = dislike very much; and 1 = dislike extremely).

a,b Means within a row in each cut with different letters are different at p<0.05.

**Table 6:** Correlation coefficients (*r*) between fat level groups and selected meat quality traits in pork bellies

Items	Fat level		

	Low fat	Medium fat	High fat
Live weight	0.119	0.232	0.830^*^
Dressing	−0.887	0.044	0.843^*^
Skin yield	−0.163	0.273	0.396
Trimmed fat yield	−0.799	−0.121	0.920^*^
Bone yield	0.340	0.050	0.390
Belly yield	−0.366	0.327	0.835^*^
C14:0	−0.342	0.985	−0.643
C16:0	−0.299	−0.277	0.397
C16:1n7	0.500	0.500	0.400
C18:0	0.269	0.271	0.398
C18:1n9	0.373	0.687	0.836^*^
C18:2n6	0.691^*^	0.572	−0.281
C18:3n3	0.912^*^	0.611	0.101
C20:1n9	−0.444	−0.554	0.198
C20:4n6	0.634	0.497	−0.564
C22:4n6	0.693	0.371	−0.277
SFA	−0.079	−0.224	0.103
UFA	0.079	0.024	−0.203
MUFA	0.360	0. 372	0.237
PUFA	0.795^*^	0.470	−0.275
n6/n3	0.485	−0.040	−0.345
MUFA/SFA	0.245	−0.189	−0.456
PUFA/SFA	−0.772^*^	0.377	−0.305
Fat	−0.873	0.015	0.859^*^
Moisture	0.873^*^	0.147	−0.930^*^
Protein	0.852^*^	0.027	−0.879^*^
Collagen	0.813^*^	0.097	−0.911^*^
CIE L*	−0.386	0.346	0.539
CIE a*	−0.470	0.299	−0.429
CIE b*	−0.838	−0.054	0.892
Chroma	−0.478	0.499	−0.201
Hue angel	−0.940^*^	0.576	0.176
Cooking loss	0.451	0.236	−0.866
Overall sensorial color	−0.500	−0.500	0.860^*^
Flavor	−0.738	−0.215	0.953^*^
Juiciness	−0.779	−0.154	0.933^*^
Tenderness	−0.733	−0.223	0.956^*^
Overall acceptance	−0.701	−0.267	0.968^*^

SFA, saturated fatty acids; UFA, unsaturated fatty acids; MUFA, mono unsaturated fatty acids; PUFA, poly unsaturated fatty acids.

* p<0.05.
